# Combining mindfulness and cognitive training in children with attention deficit hyperactivity disorder: study protocol of a pilot randomized controlled trial (the NeuroMind study)

**DOI:** 10.3389/fpsyg.2024.1291198

**Published:** 2024-02-07

**Authors:** Tania Badia-Aguarón, Estíbaliz Royuela-Colomer, Vanessa Pera-Guardiola, Pere Vergés-Balasch, Ausiàs Cebolla, Juan V. Luciano, Joaquim Soler, Albert Feliu-Soler, Anna Huguet Miguel

**Affiliations:** ^1^Department of Basic, Developmental and Educational Psychology, Faculty of Psychology, Autonomous University of Barcelona, Barcelona, Spain; ^2^Psychological Research in Fibromyalgia and Chronic Pain (AGORA Research Group), Parc Sanitari Sant Joan de Déu, Barcelona, Spain; ^3^CIBER of Epidemiology and Public Health (CIBERESP), Madrid, Spain; ^4^Child and Adolescent Mental Health Service Sant Joan de Déu Terres de Lleida, Lleida, Spain; ^5^Department of Psychology, University of Lleida, Lleida, Spain; ^6^Institut de Recerca Biomèdica de Lleida, Lleida, Spain; ^7^Department of Personality, Assessment and Psychological Treatments, University of Valencia, Valencia, Spain; ^8^CIBER of Obesity and Nutrition (CIBEROBN), Madrid, Spain; ^9^Department of Clinical and Health Psychology, Faculty of Psychology, Autonomous University of Barcelona, Barcelona, Spain; ^10^Servei de Psiquiatria, Hospital de la Santa Creu i Sant Pau, Barcelona, Spain; ^11^Department of Psychiatry and Forensic Medicine, Autonomous University of Barcelona, Barcelona, Spain; ^12^CIBER of Mental Health (CIBERSAM), Madrid, Spain; ^13^Department of Medicine, University of Lleida, Lleida, Spain; ^14^Sant Joan de Déu Research Institute (IRSJD), Esplugues de Llobregat, Spain

**Keywords:** ADHD, children, executive functions, mindfulness, cognitive training, randomized controlled trial, effectiveness, feasibility

## Abstract

**Introduction:**

Attention Deficit Hyperactivity Disorder (ADHD) has a global mean prevalence of 5%. Cognitive Training (CT) and Mindfulness-Based Interventions (MBIs) have shown promising results in managing ADHD symptoms, but they are not its Treatment-As-Usual (TAU). The NeuroMind Study aims to evaluate the preliminary effectiveness and feasibility of three interventions: Mindfulness for Health (M4H), CT using the NeuronUP® platform (CT), and a combination of both, Mindfulness Cognitive Training (MCT). There is empirical evidence supporting the effectiveness of the M4H and NeuronUP® platform; however, this study explores for the first time the effectiveness of MCT and CT, as well as the integration of M4H into TAU. The objectives of this 5-month Randomized Controlled Trial (RCT) are: (1) To analyze the preliminary effectiveness and feasibility of M4H, CT or a combination of both (MCT) added to TAU for children with ADHD; (2) To evaluate the role of psychological process variables (mindfulness and emotional regulation) as mediators of 5-month follow-up clinical outcomes; (3) To preliminarily explore whether specific sociodemographic and clinical characteristics can predict the short-and medium-term clinical response to the specific treatments.

**Methods and analysis:**

Participants will be 120 children (7 to 12 years) with ADHD recruited at Child and Adolescent Mental Health Service (CAMHS) Sant Joan de Déu Terres de Lleida (Spain) randomly allocated to one of the four study arms: TAU vs. TAU + CT vs. TAU + M4H vs. TAU + MCT. An assessment to collect ADHD symptoms, Executive Functions (EF), comorbid symptoms and global functioning will be conducted pre-intervention, post-intervention (2 months after baseline) and at the 5-month follow-up. Linear mixed models and mediational models will be computed.

**Discussion:**

If the preliminary effectiveness and feasibility of the MCT are demonstrated, this study could be a preliminary basis to do a full RCT with a larger sample to definitively validate the intervention. The MCT could be applied in clinical practice if it is definitively validated.

**Clinical trial registration:**ClinicalTrials.gov, identifier, NCT05937347. https://clinicaltrials.gov/study/NCT05937347?locStr=Spain&country=Spain&cond=ADHD&intr=Mindfulness&rank=1.

## Introduction

Attention Deficit Hyperactivity Disorder (ADHD) is a neurodevelopmental disorder that typically begins in childhood and can persist into adulthood and is characterized by persistent patterns of inattention, hyperactivity, and impulsivity that are not age-appropriate and interfere with a child’s daily functioning and development [American Psychiatric Association (APA), 2014]. Its neurocognitive profile shows some deficits, mainly in executive functions (EF), including alterations in verbal fluency, working memory, cognitive flexibility, inhibition, decision-making, memory, planning, reaction time, selective attention and vigilance (Pievsky and McGrath, 2018). Furthermore, children with ADHD usually present other comorbid disorders such as anxiety and mood disorders, Oppositional Defiant Disorder, Conduct Disorder, Learning Disorder, Obsessive-Compulsive Disorder, Autism Spectrum Disorder [American Psychiatric Association (APA), 2014; Nigg et al., 2020; Jones et al., 2022]. In adulthood, ADHD symptoms that persist cause a negative functioning (Cherkasova et al., 2022) but its persistence depends on an early pharmacological treatment, educational support and neuropsychological intervention in the childhood (Varrasi et al., 2023).

ADHD has a mean prevalence of 5% [American Psychiatric Association (APA), 2014; Sayal et al., 2018] with a 2:1 male-to-female ratio [American Psychiatric Association (APA), 2014; Magnus et al., 2023], being the inattentive and the combined (inattentive and impulsive) subtypes the most commonly observed (Leffa et al., 2022). Evidence suggests that ADHD has a hereditary component—as it is more prevalent among children of parents with ADHD [American Psychiatric Association (APA), 2014; Uchida et al., 2023]—and several environmental factors have also been determined [e.g., prenatal exposure to certain substances; pregnancy and birth complications, specific family interaction patterns among others; American Psychiatric Association (APA), 2014; Kian et al., 2022].

The Treatment-As-Usual (TAU) for children with ADHD are, in mild cases, mainly non-pharmacological (e.g., psychoeducation and behavioral interventions); in moderate and more severe cases, the gold-standard treatment is usually psychopharmacological in nature, including stimulants (e.g., amphetamine and methylphenidate)—applicable in 70% of cases—and non-stimulant drugs (e.g., atomoxetine, bupropion, clonidine and guanfacine; Ministerio de Salud, Servicios Sociales e Igualdad, 2018; National Institute for Health and Care Excellence, 2018; Magnus et al., 2023).

Psychopharmacology, although considered an efficient treatment with large effect sizes in short term trials (Mechler et al., 2022) as long it is taken, has remarkable adverse effects (e.g., weight loss, sleep disturbance, irritability, tachycardia, headache), and dose increases along treatment are necessary to maintain the full efficacy of the drugs (Caye et al., 2019). In this regard, an approach combining non-pharmacological and pharmacological treatments is recommended since it is considered efficacious at lower medication doses (Caye et al., 2019; Wolraich et al., 2019; Drechsler et al., 2020) and Türk et al. (2023) find that there is a lack of research on combined treatments.

Among non-pharmacological treatments, psychoeducation is offered as a first-option approach and is aimed at increasing understanding of the disorder and providing tips for better management of the symptoms (Drechsler et al., 2020; López-Pinar et al., 2020). When it comes to behavioral treatments for ADHD, various approaches have been explored, such as Cognitive-Behavioral Therapy (CBT) and social skills training have been found efficacious, particularly in the short term, on behavior, parenting skills, child–parent relationship, but its effects in ADHD symptoms are inconsistent when only blinded assessments are considered (Wolraich et al., 2019; Drechsler et al., 2020). Additionally, other non-pharmacological treatments are available, including physical activity, which is effective as a complementary treatment, or neurofeedback, which has shown effectiveness, albeit at a higher cost and with potential side effects (Wolraich et al., 2019; Drechsler et al., 2020). Furthermore, dietary modifications have also demonstrated some effectiveness, although the improvement tends to be of small magnitude (Wolraich et al., 2019; Drechsler et al., 2020). Finally, Cognitive Training (CT) and Mindfulness-Based Interventions (MBIs) have also shown promising results in managing ADHD symptoms (Caye et al., 2019).

CT is a therapeutic approach focused on cognitive functioning and aimed at improving the individual’s capacity to process and use incoming information to allow increased functioning in everyday life (Bahar-Fuchs et al., 2013; Cruz-González et al., 2021). CT consists of repetitive and increasing difficulty exercises focused on one or multiple cognitive domains (Caye et al., 2019; Drechsler et al., 2020). There is compelling evidence to suggest that CT can be effective in improving symptoms of ADHD in children, particularly when the training targets multiple cognitive domains instead of solely focusing on a single domain (Caye et al., 2019; Drechsler et al., 2020). CT has demonstrated positive outcomes by enhancing EF in children with ADHD. Recent studies have shown short-term improvements in various EF components, including attention, working memory, processing speed, cognitive flexibility, planning, and reasoning (Cortese et al., 2015; Caye et al., 2019; Drechsler et al., 2020; Veloso et al., 2020). Furthermore, a recent meta-analysis indicates that improved working memory after CT can be sustained over the long term (Westwood et al., 2023). The CT is typically presented to patients through games and can be administered through paper-and-pencil or computerized formats (Bahar-Fuchs et al., 2013; Caye et al., 2019). Computerized CT can be done at home so that it allows more adherence to the intervention, avoiding displacements and providing motivational environments (Maresca et al., 2020), and there are many CTs electronic interfaces available whose efficacy in ADHD has been studied (Fernández-Daza, 2019). CT could be a good intervention to adjunct to TAU in children with ADHD because it could enhance the improvement of ADHD symptoms through the improvement of the EF (Cortese et al., 2015; Caye et al., 2019; Drechsler et al., 2020; Veloso et al., 2020).

MBIs encompass education and practices designed to enhance focus on the present moment, foster decentering, and encourage an open orientation to experience (Bulzacka et al., 2018; Drechsler et al., 2020). They aim to cultivate qualities such as joy, compassion, wisdom, and equanimity, and improve attentional, emotional, and behavioral self-regulation. Participants engage in sustained intensive training, experiential inquiry-based learning processes, and exercises that promote understanding (Crane et al., 2017). Although MBIs have demonstrated significant benefits across various pathologies in both adults and children, a recent large-scale mindfulness project, “MYRIAD,” shows that most the youth of 9–18 years do not engage with mindfulness training and that it might only work for children under some conditions (Burrows, 2022). In the Spanish context, the GrowingUp Breathing Program for children of 7–12 years in the school context has been tested in an RCT with a sample of 307 children compared to their usual curriculum with positive outcomes with significant improvements pre-post that maintained at follow-up in emotional regulation and in the ability to reduce their anxiety levels (García-Rubio, 2021).

Concerning ADHD symptomatology, a recent meta-analysis including samples of adults and children found that these approaches had substantial effects in reducing ADHD symptoms (Xue et al., 2019). Additionally, in adults with ADHD, a systematic review has shown that MBIs improve EF and emotional dysregulation (Poissant et al., 2019). MBIs have also shown positive outcomes in children and youth. A review of 33 RCTs highlighted that MBIs could be useful for improving mental health and well-being in youth by also reducing levels of depression and anxiety (Dunning et al., 2019). Another systematic review of 12 RCTs involving children and youth with ADHD demonstrated that MBIs led to reductions in ADHD symptoms, externalizing behavior problems, internalizing behavior problems, and parental stress within a timeframe of 1 to 6 months (Lee et al., 2022). Moreover, in MBIs targeting children with ADHD, the active involvement of parents has shown better results (Chan et al., 2018). In an RCT of an MBI involving 103 children with ADHD and their families, has been observed a significant improvement of ADHD symptoms post-intervention in addition to TAU, compared to TAU only, of which the reduction of hyperactivity and impulsivity has been maintained at 6-month follow-up. In parents, it has been observed an improvement in their own ADHD symptoms, well-being and mindful parenting at post-intervention compared to TAU, of which their ADHD symptoms and mindful parenting maintained at follow-up, and appears an improvement in self-compassion (Siebelink et al., 2022).

In the Spanish context, the Mindfulness for Health (M4H) for children with ADHD has been tested in an RCT with a sample of 72 children compared to TAU with positive outcomes in attention, working memory, impulsivity, frustration tolerance, anxiety, affective symptomatology, emotional regulation and quality of life (Huguet et al., 2019) and with a sample of 116 children compared to TAU with positive outcomes in ADHD symptoms; EF, with large effect size in working memory and planning; externalizing symptoms and global functioning (Huguet, 2021). All these findings underscore the broad-ranging benefits of MBIs in various mental health conditions for adults and children and suggest that MBIs hold promise as effective interventions for addressing ADHD symptoms and improving overall well-being in children with ADHD.

As explained above, ADHD is characterized by difficulties in self-regulation, attention, and impulse control, along with emotional difficulties such as anxiety, depression, and poor emotional regulation. In this regard, CT focuses on improving EF, which includes skills like working memory, cognitive flexibility, and attention control. CT aims to strengthen these fundamental cognitive abilities through specific exercises and tasks. Simultaneously, MBIs have proven useful in individuals characterized by high emotional dysregulation and impulsivity (Soler, 2014, 2016; Carmona i Farrés et al., 2019), even using short-time practices. Emotion regulation and dispositional mindfulness are two interrelated psychological constructs. Some studies (Baltruschat et al., 2021) suggest that the neural bases behind these are closely associated. In this regard, combining CT and mindfulness training may be particularly effective in patients with ADHD due to the interconnectedness between cognitive and emotional processes. By enhancing EF through CT, improvements in attention, memory, and self-control can be achieved. At the same time, MBI can provide tools to manage the emotional challenges associated with ADHD, thereby reducing emotional distraction and enhancing emotional regulation. In summary, combining cognitive and mindfulness training in ADHD treatment may increase effectiveness by harnessing the benefits of simultaneously targeting cognitive deficits and emotional dysregulation (Soler, 2014, 2016; Carmona i Farrés et al., 2019).

In this line, a recent article suggests the potential benefits of the combination of Computerized CT and MBI in depression, considering that CT may improve EF affected in ADHD and MBIs target emotional regulation deficits, so the combination of both may cause a faster and more robust clinical improvement (Bursky et al., 2022). In this regard, Computerized CT may be a highly applicable approach due to smartphone use and MBIs may provide tools that patients can use in their daily living (Bursky et al., 2022). Another recent article highlights the benefits of this combination of interventions in the cognition, mood and quality of life of older adults (Sanchez-Lara et al., 2023). A similar argument is reasonable to apply to ADHD. To date, there is no study on the combination of CT and MBIs in treating ADHD in children. By addressing the cognitive and emotional aspects of ADHD, this combination theoretically may offer a comprehensive and more effective approach to improving symptoms and associated ADHD difficulties.

The objectives of the NeuroMind study are (1) to analyze the preliminary effectiveness (short-and medium-term) and feasibility of adding the Mindfulness for Health (M4H) program, a computerized (online) CT program or a combination of both (Mindfulness Cognitive Training; MCT) to the TAU for children with ADHD; (2) to explore whether the effect sizes of the primary outcome of ADHD are larger in M4H and MCT programs than in CT program; (3) to evaluate the role of psychological process variables considered to be potential mediators of the interventions from a theoretical point of view (i.e., mindfulness, emotional dysregulation); (4) For the sake of personalized treatment in ADHD, the current study has also been designed to preliminarily explore whether specific baseline clinical characteristics can serve as predictive factors for the short-and medium-term clinical response to the specific treatments being evaluated.

The possible demonstration of the preliminary effectiveness in the short-and medium-term and the feasibility of the M4H, CT and MCT programs in this NeuroMind study and the probability to obtain larger effects sizes for M4H and MCT programs in the primary outcome of ADHD could be a preliminary basis for conducting a full RCT with a larger sample in the future, in order to definitively validate these all three interventions. If the M4H, CT and MCT programs are definitely validated they should be incorporated into clinical practice.

## Materials and methods

### Study design

This RCT protocol was developed following the Standard Protocol Items: Recommendations for Interventional Trials (SPIRIT; Chan et al., 2013) and was recorded in the ClinicalTrials.gov trial register on 5 July 2023 (Trial registration number: NCT05937347). The Consolidated Standards of Reporting Trials 2010 (CONSORT; Schulz et al., 2010) and the Consolidated Health Economic Evaluation Reporting Standards (CHEERS; Husereau et al., 2013) will be followed.

**Figure 1:**
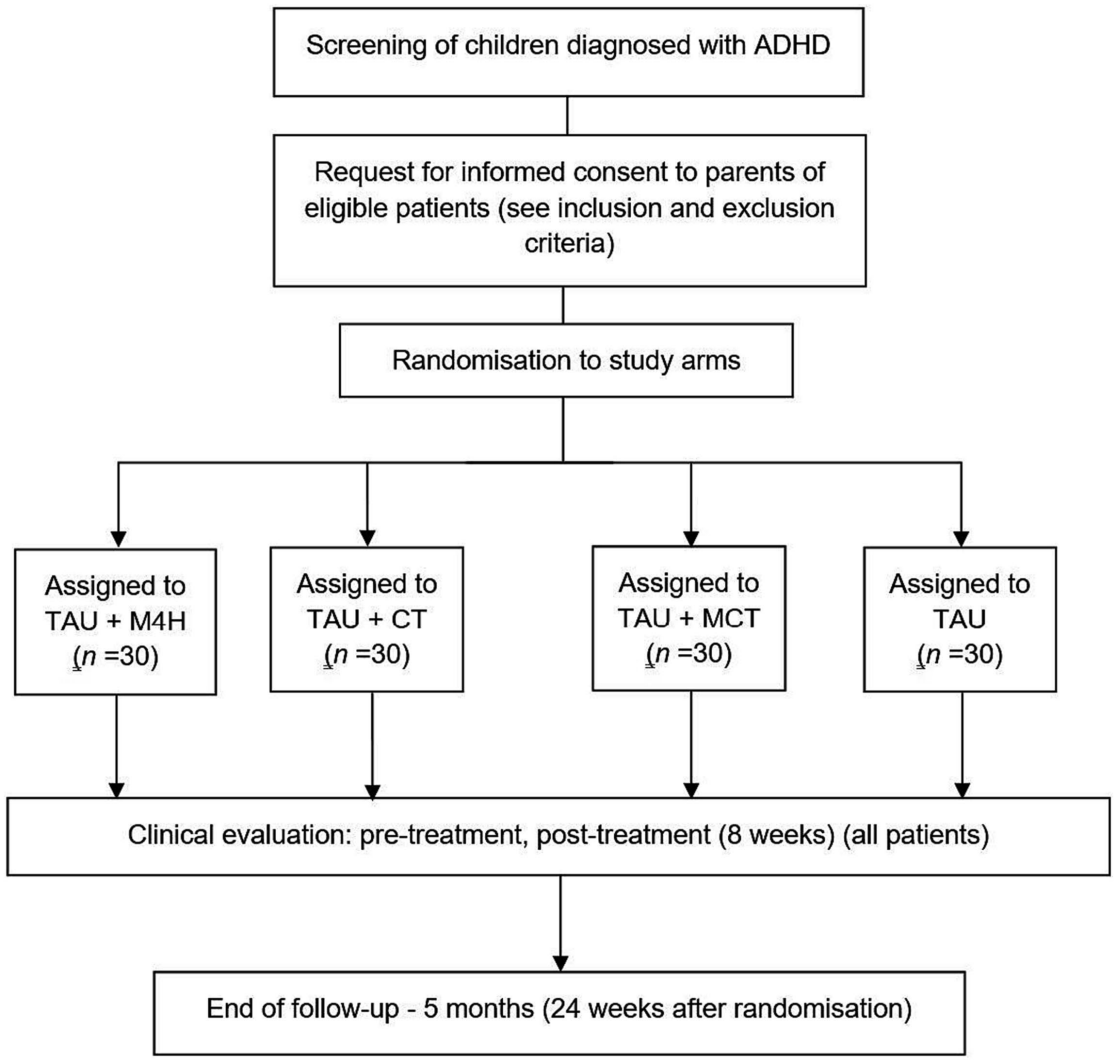
Flowchart of the NeuroMind study based on the Consolidated Standards of Reporting Trials guidelines. M4H, Mindfulness for Health; CT, Cognitive Training; MCT, Mindfulness for Health plus NeuronUP; TAU, Treatment-As-Usual.

This study is a 5-month, parallel-group and single-blind RCT aimed at preliminarily assessing the medium-term effectiveness and feasibility of (1) a validated MBI for ADHD (Mindfulness for Health; M4H; Hospital Sant Joan de Déu, 2019) including parent participation, (2) a Computerized CT program through NeuronUP®, and (3) the combination of both approaches (M4H; CT; MCT) in addition to TAU in a sample of children with ADHD, compared to TAU alone. Assessments will be conducted at baseline, post-intervention (2 months after baseline), and at 5-month follow-up. See the study flowchart in Figure 1.

### Study setting

The trial will be single-center and will be conducted at CAMHS Sant Joan de Déu Terres de Lleida (Lleida, Spain), although part of the intervention will be completed off-site using a secure digital platform: NeuronUP®.

### Eligibility criteria

The inclusion criteria for the present study are (a) age between 7 and 12 years; (b) primary diagnosis of ADHD according to the DSM-5 criteria by a psychologist and confirmed by the Kiddie-Schedule for Affective Disorders and Schizophrenia Present and Lifetime Version DSM 5 (K-SADS-PL-5; Kaufman et al., 1997) Spanish Version; (c) ADHD medication dose stable in the last 2 months or an informed decision on not taking ADHD medication during the study; (d) Spanish language; (e) Availability to meet all visits; (f) Understanding the conditions of the study and signing the informed consent by parents or legal guardians. The exclusion criteria for the present study are: (a) Diagnosis of Autism Spectrum Disorder (ASD) according to the DSM-5 criteria by a psychologist or confirmed by the Social Communication Questionnaire-Form B (SCQ-Form B; Rutter et al., 2003); (b) Presenting psychosis, bipolar illness, active suicidality or untreated posttraumatic stress disorder (checked by the K-SADS-PL-5 Spanish Version); (c) Intelligence Quotient (IQ) ≤ 80, checked by the Kaufman Brief Intelligence Test (K-BIT); (d) Receiving psychological or psycho-educational treatment in the last 2 months or disagreement of the parents in not seek it during the study; (e) Participation in an MBI in the past year; (f) Participation in another clinical trial at the same time.

### Interventions

#### Mindfulness for health program

The Mindfulness for health program (M4H) is a Spanish MBI of 8-week sessions of 1.25 h and daily homework for children with ADHD from 7 to 12 years old based on evidence-based techniques from previous MBIs (Hospital Sant Joan de Déu, 2019). M4H was designed to provide a tool for mental health specialists to reduce ADHD symptoms, improve emotional regulation, promote self-awareness and self-control and improve the quality of life of children with ADHD and their families (Hospital Sant Joan de Déu, 2019). Since MBIs that involve parents have better results (Chan et al., 2018; Siebelink et al., 2022), we add 3-week sessions of 1.25 h for parents. We also add the submission of a sheet with the description of the entire M4H program at the beginning of the intervention, and a sheet after each session with a summary of its content and the description of the homework. See Table 1 for more details.

**Table 1:** Outline of the M4H sessions.

Session	Children with ADHD	Parents	Homework
1	Group cohesion	Mindfulness psychoeducation	My breathing stones (6 days practice of 3 min)
	Mindfulness psychoeducation	Mindfulness of the breath	
	Mindfulness of the breath	Home practice recommendations	
2	Self-observation		My breathing stones (6 days practice of 3 min)
			Self observation (2 days practice)
	Mindfulness of the breath		Emotional sheet
3	Body scan		My breathing stones (6 days practice of 4 min)
	Mindful walking		Mindful put on and take off shoes (6 days)
4	Mindful body exercises	Reinforcing home practice	My breathing stones (6 days practice of 5 min)
		Mindfulness of the breath	
	Mindfulness of the breath	Body scan	Mindful coloring mandala (6 days)
		Mindful parenting	
5	Mindful eating		My breathing stones (6 days practice of 6 min)
			Mindful eating (6 days)
6	Emotional awareness		My breathing stones (6 days practice of 7 min)
			Anger awareness (6 days)
7	Difficulties awareness		My breathing stones (6 days practice of 9 min)
			Difficulties awareness (6 days)
8	Emotional awareness	Practicing mindfulness after group ending	My breathing stones (6 days practice of 11 min)
	Qualities awareness	Emotional awareness	Emotional awareness (1 day)
	Closing of the group	Closing of the group	

#### Cognitive therapy

Cognitive therapy (CT) was delivered through the interface NeuronUP®, which is designed as a computerized CT program of 8-week sessions of 60 min (10 min per day) of specific non-verbal activities for children, to improve the cognitive domains mainly affected in ADHD (Pievsky and McGrath, 2018). CT also includes on-site 3-week sessions of 30 min of psychoeducation and discussing the home practice with children and parents. NeuronUP® reports the number of times the child practices and 5-min follow-up calls are made once every 2 weeks by an experienced psychologist to resolve doubts and to motivate the practice. NeuronUP® is a Spanish CT electronic interface created in 2012 (NeuronUP S.L., 2023). Its objective is to provide a tool for professionals to plan their sessions and programs. NeuronUP® can be available from wherever through a computer, tablet or phone with an internet connection. It is available in Spanish, English, French, Portuguese and Catalan. It contains many activities specifically designed to improve the cognitive domains and the activities of the daily living of the child or adult population with different cognitive impairments or mental illnesses (NeuronUP S.L., 2023). NeuronUP® has some ongoing studies and few published studies. Regarding its published studies, we find, for example, that its exercises improve global cognition and everyday memory in older adults with mild cognitive impairment (Mendoza-Laiz et al., 2018; Cruz-González et al., 2021), and improvements in attention and working memory in children and adolescents with Intellectual Disabilities (ID; Torra, 2022). However, there are no published results of its effectiveness in ADHD yet, but there are other ongoing studies in this regard (NeuronUP S.L., 2023). See Table 2 for more details.

**Table 2:** Outline of the CT program.

Week	Online	On-site
1	Sustained attention: Hit the ball (5 min)	CT psychoeducation
		NeuronUP® demonstration
	Working memory: Who raised their hand? (5 min)	Online practice recommendations
2	Selective attention: Seek and find (5 min)	-
	Processing speed: Hungry animals (5 min)	
3	Shifting: Waiter in action (5 min)	-
	Animated labyrinth (5 min)	
4	Flexibility: The best organizer (5 min)	Reinforcing online practice
	Order on the farm (5 min)	
5	Inhibition: The first dog (5 min)	-
	Beat the monster (5 min)	
6	Reasoning: Connected drawings (5 min)	-
	Step by step (5 min)	
7	Time estimate: How much time do you need? (10 min)	-
8	Planning: Escape the labyrinth (5 min)	Thinking on online practice
	Prepare the school backpack (5 min)	Closing of the group

#### MCT

MCT is an intervention composed of the content of M4H and CT interventions with an adaptation of the parent sessions and a reduction of the number of repetitions of the different home tasks of M4H (3 days per week) and of the CT sessions (30 min, 10 min per day). See Table 3 for more details.

**Table 3:** Outline of the MCT Sessions.

Session	Children with ADHD	Parents	Homework
1	Group cohesion	Mindfulness and CT psychoeducation	My breathing stones (3 days practice of 3 min)
	Mindfulness psychoeducation	Mindfulness of the breath	Sustained attention: Hit the ball (3 days practice of 5 min)
	Mindfulness of the breath	Home practice recommendations	Working memory: Who raised their hand? (3 days practice of 5 min)
2	Self observation		My breathing stones (3 days practice of 3 min)
			Self observation (1 day practice)
			Emotional sheet
	Mindfulness of the breath		Selective attention: Seek and find (3 days practice of 5 min)
			Processing speed: Hungry animals (3 days practice of 5 min)
3	Body scan		My breathing stones (3 days practice of 4 min)
			Mindful put on and take off shoes (3 days)
	Mindful walking		Shifting: Waiter in action (3 days practice of 5 min)
			Animated labyrinth (3 days practice of 5 min)
4	Mindful body exercises	Reinforcing home practice	My breathing stones (3 days practice of 5 min)
		Mindfulness of the breath	Mindful coloring mandala (1 day)
	Mindfulness of the breath	Body scan	Flexibility: The best organizer (3 days practice of 5 min)
		Mindful parenting	Order on the farm (3 days practice of 5 min)
5	Mindful eating		My breathing stones (3 days practice of 6 min)
			Mindful eating (3 days)
			Inhibition: The first dog (3 days practice of 5 min)
			Beat the monster (3 days practice of 5 min)
6	Emotional awareness		My breathing stones (3 days practice of 7 min)
			Anger awareness (3 days)
			Reasoning: Connected drawings (3 days practice of 5 min)
			Step by step (3 days practice of 5 min)
7	Difficulties awareness		My breathing stones (3 days practice of 9 min)
			Difficulties awareness (3 days)
			Time estimate: How much time do you need? (3 days practice of 10 min)
8	Emotional awareness	Practicing mindfulness after group ending	My breathing stones (3 days practice of 11 min)
	Qualities awareness	Emotional awareness	Emotional awareness (1 day)
			Planning: Escape the labyrinth (3 days practice of 5 min)
	Closing of the group	Closing of the group	Prepare the school backpack (3 days practice of 5 min)

#### Treatment as usual

The treatment as usual (TAU) group will continue with their usual ADHD treatment except for psychological or psycho-educational continued treatment: non-treatment, psychopharmacology, psychiatric monitoring and/or psychological monitoring.

Possible medication changes in participants during the study will be assessed for further consideration in the statistical analyses, or consideration will be given to excluding the case. After completion of the study, participants in the TAU group will be offered the possibility of CT intervention.

All active arms (i.e., M4H group, CT group, and MCT group), apart from the treatment described above, also receive TAU.

### Study measures

Assessment of outcome and process measures will be carried out at baseline, at post-intervention (2 months) and at 5-month follow-up after starting the trial by evaluators with broad experience in applying the study measures. All of the study instruments will be administered to all participants through paper-and-pencil tests. The Mindfulness-Based Interventions: Teaching Assessment Criteria (MBI:TAC) is the only test that will be completed during intervention, and it will be only completed at MCT and M4H groups The MCT, M4H and CT parent and children satisfaction questionnaires will be only completed at post-intervention at intervention groups. See Table 4 for a summary of assessments.

**Table 4:** Summary of the assessments in the RCT.

	Baseline	Post-intervention (2 months)	Follow-up (5 months)
Sociodemographic and clinical data	X		
Screening instruments			
For parents			
SCQ-Form B	X		
K-SADS-PL-5	X		
For children			
K-BIT	X		
Primary outcome			
ADHD-RS-IV	X	X	X
Secondary outcomes			
For parents			
CPRS-R	X	X	X
For children			
ENFEN	X	X	X
SCARED	X	X	X
Conners CPT 3	X	X	X
For psychologists			
CGAS	X	X	X
CGI-S	X	X	X
Process outcomes	X	X	
For children			
MAAS-C	X	X	X
For parents			
CBCL	X	X	X
Other measures		X	
For parents	X		
CEQ		X	
Satisfaction questionnaires		X	
Perceived adverse effects of the interventions		X	
For children			
Satisfaction questionnaires		X	
Adherence to home practice		X	
For psychologists			
MBI:TAC		X	

SCQ-Form B, Social Communication Questionnaire-Form B; K-SADS-PL-5, Kiddie-Schedule for Affective Disorders and Schizophrenia Present and Lifetime Version DSM 5; K-BIT, Kaufman Brief Intelligence Test; ADHD-RS-IV, ADHD Rating Scale-IV; CPRS-R, Conners Parents Revised Scale; ENFEN, Neuropsychological Assessment of Executive Functions in Children; SCARED, Screen for Child Anxiety Related Emotional Disorders; Conners CPT 3, Conners Continuous Performance Test; CGAS, Children’s Global Assessment Scale; CGI-S, Clinical Global Impression Scale-Severity; MAAS-C, The Mindful Attention Awareness Scale Adapted for Children; CBCL, Child Behavior Checklist; CEQ, Adapted version of Credibility/Expectancy Questionnaire; MBI:TAC, Mindfulness-Based Interventions: Teaching Assessment Criteria.

Socio-demographic and clinical data will be collected at baseline: age, sex, education, parents’ or legal guardians’ education and employment and personal and family medical history; as well as the presence of psychopharmacological treatment (name and dosage), the current and previous diagnosis and comorbidities. The psychopharmacological treatment will also be collected at post-intervention and at the 5-month follow-up in case there were changes.

### Screening instruments

#### Screening instruments for parents

Social Communication Questionnaire-Form B (SCQ-Form B; Rutter et al., 2003) Spanish version (Rutter et al., 2019): Questionnaire for parents on current social communication of the children to ASD detection. The questionnaire has 40 items with yes/no questions (*presence of symptoms* with a score of 1/ *absence of symptoms* with a score of 0) and a risk cutoff of 15. The Spanish version of the SCQ is acceptable (α = 0.81; Rutter et al., 2003, 2019).

Kiddie-Schedule for Affective Disorders and Schizophrenia Present and Lifetime Version DSM 5 (K-SADS-PL-5; Kaufman et al., 1997) Spanish Version (de la Peña et al., 2018): Semi-structured interview for parents for the detection of Affective Disorders and Schizophrenia that contains the primary symptoms of each disorder. The psychologist produces a summary for each symptom (*no information* with a score of 0/*absent* with a score of 1/*subthreshold* with a score of 2/ *threshold* with a score of 3). The original and the Spanish versions of the K-SADS-PL-5 obtained good inter-rater results, with a Kappa index between 0.76 and 1 (Kaufman et al., 1997; de la Peña et al., 2018).

#### Screening instruments for children

Kaufman Brief Intelligence Test (K-BIT; Kaufman and Kaufman, 1990) Spanish version (Kaufman and Kaufman, 1990): General Intelligence test for children and adults (4–90 years) applied in patients. It is composed of two subtests: Vocabulary (verbal IQ), which comprises 82 items to assess verbal skills related to school learning, language knowledge, information flow and the level of verbal conceptualisation; and Matrices (non-verbal IQ), composed of 48 elements with drawings and figures, to assess the capacity for logical non-verbal and spatial reasoning. The total IQ score is obtained from the sum of both typical scores. The Spanish version demonstrated excellent concurrent, construct validity and test–retest reliability coefficients (Kaufman and Kaufman, 1990).

#### Primary outcome

ADHD Rating Scale-IV (ADHD-RS-IV; DuPaul et al., 1998) Parents version: Questionnaire for parents with a Likert scale response (0–3) that includes an item for each of the 18 symptoms listed in the DSM-5 diagnostic criteria for ADHD: 9 for inattention, 6 for hyperactivity and 3 for impulsivity. Six or more of the inattention items and/or 6 or more hyperactivity-impulsivity items must be fulfilled to diagnose ADHD. Depending on the symptoms met, the inattentive, hyperactive–impulsive or combined subtype is specified. The internal consistency of the Spanish version is good (α = 0.86; DuPaul et al., 1998; Vallejo-Valdivielso et al., 2019).

### Secondary outcomes

#### Secondary outcomes for parents

Conners Parents Revised Scale (CPRS-R; Conners et al., 1998) Short Version: Scale for parents of 28 items that assess ADHD and disruptive behavior through 4 subscales: oppositional, inattention, hyperactivity-impulsivity and ADHD index. Each item is assessed by a Likert scale of 4 points ranging from 0 to 3 points (*it is not true* with a score of 0/*sometimes it is true* with a score of 1/*many times it is true* with a score of 2/*always it is true* with a score of 3). Higher scores indicate greater severity of symptoms or risk of meeting ADHD diagnostic criteria. Internal consistency coefficients of the Spanish version range from 0.89 to 0.93 (Farré-Riba, 1997; Conners et al., 1998).

#### Secondary outcomes for children

Neuropsychological Assessment of Executive Functions in Children (ENFEN; Portellano et al., 2009): Instrument battery applied in Spanish children (6–12 years) that assesses the different EF, composed of 4 tests: verbal fluency, trail construction, construction with rings and resistance to interference. The verbal fluency test assesses the language domain and the semantic memory. Trails test assesses attention and cognitive flexibility. The rings test assesses working memory and planning. The interference test assesses the inhibition ability. Higher scores indicate greater performance in the evaluated function, except in the rings tests, which works backwards. Internal consistency coefficients range from 0.81 to 0.96 (Portellano et al., 2009).

Screen for Child Anxiety Related Emotional Disorders (SCARED; Birmaher et al., 1999): It allows the assessment of anxious symptomatology and is answered by patients with an intraclass correlation coefficient range from 0.71 to 0.90 in the Spanish version (Birmaher et al., 1999; Canals et al., 2012).

Conners Continuous Performance Test (Conners CPT 3; Conners, 2014): The CPT 3 is applied in children from 8 years and it assesses sustained attention, selectivity attention and inhibition across a computerized task of continuous execution of 14 min in which children must press a button each time a letter except X appears on the screen. The CPT 3 reports on 4 indexes: inattentiveness, sustained attention, vigilance and impulsivity. The reliability coefficients (Cronbach’s alpha) range from 0.93 and 0.96 (Conners, 2014).

#### Secondary outcomes for psychologists

Children’s Global Assessment Scale (CGAS; Shaffer et al., 1983): The scale answered by the psychologist allows qualifying the general functioning in youth up to 18 years. The scores range from 1 to 100, with higher scores for greater performance. The internal consistency of the Spanish version is good (α = 0.85; Shaffer et al., 1983; Ezpeleta et al., 1999).

Clinical Global Impression Scale-Severity (CGI-S; Guy, 1976): It is a scale completed by the psychologist that allows qualifying the illness severity as an indicator of clinical global impression in the pediatric and adult population. The scores range from 0 to 7, where higher scores mean a worse clinical global impression (Guy, 1976).

### Process outcomes

#### Process outcomes for children

The Mindful Attention Awareness Scale Adapted for Children (MAAS-C; Lawlor et al., 2014): It is a scale that assesses mindfulness, is completed by the patient, and has 15 items. Each item is assessed by a Likert scale of 6 points ranging from 1 to 6 points (*Almost always* with a score of 1/ *very common* with a score of 2/ *quite often* with a score of 3/ *something rare* with a score of 4/ *very rare* with a score of 5/ *almost never* with a score of 6). It has a high internal consistency (Cronbach’s alpha = 0.84; Lawlor et al., 2014). The effect of interventions on the process variable dispositional Mindfulness is assessed and through that variable the mediating role of the effects of the interventions on the main variables in the medium-term (5-month follow-up) is analyzed.

#### Process outcomes for parents

Child Behavior Checklist (CBCL; Achenbach and Rescorla, 2001) family version: It is a broad-spectrum scale answered by parents for assessing externalizing and internalizing pathology. It measures dysfunctional behavior and anxiety. The variable referring to emotional dysregulation: Variable understood as poor modulation of emotional responses. Score formed by the sum of the standardized values (SV) for the anxiety/depression scale, the attention problems scale and the aggressive behavior scale. SV ≤ 179 indicates adequate emotional regulation; SV ≥ 180 but <210 indicates mild–moderate emotional dysregulation; SV ≥ 210 indicates severe dysregulation (Achenbach and Rescorla, 2001). The internal consistency coefficients in a Spanish sample range from 0.71 to 0.90 (Amador et al., 2006). Moreover, through the emotional dysregulation variable the mediating role of the effects of the interventions on the main variables in the medium-term (5-month follow-up) is analyzed.

### Other measures

#### Other measures for parents

Adapted version of Credibility/Expectancy Questionnaire (CEQ; Devilly and Borkovec, 2000): It is a questionnaire of 6 items answered by parents and used to assess treatment expectancy and credibility. The first part comprises three items focused on therapy credibility and three more items evaluating expectations. After finishing the treatment, the parent’s opinions regarding the treatment received by the children will also be gathered using the second part of the CEQ, which includes the same questions as in the first part but in the past tense (Devilly and Borkovec, 2000).

MCT, M4H, and CT parent satisfaction questionnaires: M4H parent satisfaction questionnaire is extracted from the M4H manual and MCT and CT questionnaires have been adapted from it. They are questionnaires of 8 items answered by parents to assess children’s and parent’s satisfaction with the intervention, its most valued aspects, and its suggestions for improvement. The questionnaires comprise 4 items assessed by a Likert scale of 5 points ranging from 1 to 5 points (*None* with a score of 1/*a little* with a score of 2/*quite* with a score of 3/*a lot* with a score of 4/*very much* with a score of 5): “Are you pleased with your child’s participation in the sessions?”; “Do you think your child is pleased to participate in the sessions?”; Would you recommend other families to participate in the sessions?; How satisfied are you with the care, help, competence and quality of the therapeutic team?; and 4 items with open-ended responses: What aspects of the sessions do you value most?; What activities do you find most useful?; Do you think the number and/or the duration of the sessions is adequate? If not, how would you improve it?; Do you have other suggestions for improvement?

An *ad hoc* item (i.e., “Have you experienced, during the course of the treatment, any unwanted symptom that you think might be directly or indirectly associated with the intervention?”) to check for the presence of perceived adverse effects (e.g., anxiety, headaches, dizziness) related to the interventions.

#### Other measures for children

MCT, M4H, and CT children satisfaction questionnaires: Like the parent questionnaires, M4H is extracted from the M4H manual and MCT and CT have been adapted from it. They are questionnaires of 7 items answered by children to assess children’s satisfaction with the intervention, its impression of improvement, its most valued aspects and its suggestions for improvement. The questionnaires comprise 4 items assessed by a Likert scale of 5 points ranging from 1 to 5 points (*None* with a score of 1/*a little* with a score of 2/*quite* with a score of 3/*a lot* with a score of 4/*very much* with a score of 5): “Did you like participating in the sessions?”; “Did participating in the sessions help you?”; “Would you like to do more sessions?”; “Would you recommend a friend to participate in the sessions?”; and 3 items with open-ended responses: “What activity did you like the most?”; “Did you learn something?”; “Would you change something?”

Adherence to Home Practice: This variable measures the extent to which participants comply with assigned home practice tasks. A logbook asking for adherence to home mindfulness practices will be administered to children every week. Adherence to cognitive training activities will be automatically recorded through the NeuronUp platform.

#### Other measures for psychologists

The Mindfulness-Based Interventions: Teaching Assessment Criteria (MBI: TAC; Floyd et al., 2023): It is a scale completed by a psychologist who is observing the mindfulness session, in order to support good-practice in Mindfulness-Based teaching, training supervision and research contexts. The scale assesses through 6 domains: Coverage, pacing and organization of session curriculum, Relational skills, Embodying mindfulness, Guiding mindfulness practices, Conveying course themes through interactive inquiry and didactic teaching and Holding the group learning environment, with a different number of items for each domain, with a total of 27 items. Each item is assessed by a Likert scale of 6 points ranging from 1 to 6 points (*Incompetent* with a score of 1/*beginner* with a score of 2/*advanced beginner* with a score of 3/*competent* with a score of 4/*proficient* with a score of 5/*advanced* with a score of 6; Crane and Kuyken, 2019). It has shown an intraclass correlation coefficient range from 0.53 to 0.69 (Floyd et al., 2023).

### Procedure

Pre-study documents have been prepared: the Ethics Committee document, study registration on ClinicalTrials.gov (Trial registration number: NCT05937347), informed consent and information sheet for participants and guardians, database and data collection booklet, and psychoeducational documents on the interventions.

Participants will be recruited by part of the psychologists from the research team by derivation from their referring psychologists at CAMHS Sant Joan de Déu Terres de Lleida by consecutive sampling, until reaching the sample needed (N = 120), registering the treatment demand.

Participants and parents/tutors will be interviewed in order to know if they meet the selection criteria, and to give the informed consent form to sign and the information sheet.

Participants will be assigned to groups through an allocation sequence generation by stratified randomization by age and ADHD subtype.^1^ There will be four recruitment waves of 7–8 participants per group, so there will be 30 participants in each wave in total.

The participants and their families will be informed about their group and the treatment will be applied in the three experimental groups (i.e., M4H, CT and MCT) added to TAU, and there will be a TAU group.

The children in the TAU group will receive the CT intervention after their 5-month follow-up assessment.

### Sample size

Results from a previous study of the group (Huguet, 2021) indicate a large effect size (*d = 1*.17) for the primary ADHD-RS-IV variable when comparing the M4H vs. TAU. Parallelly, a review and meta-analysis of RCTs about non-pharmacological interventions in children and adolescents with ADHD, shows a medium effect size of 0.64 in CT interventions (Sonuga-Barke et al., 2013). Considering these effect sizes, and according to Whitehead et al. (2016) recommendations, a minimum sample size per treatment arm of 15 would be needed considering 90% power and two-sided 5% significance in this pilot trial.

### Statistical analysis

The statistical analysis of the data will be carried out with SPSS 29 and Mplus 7.0 software. With the SPSS 29 software, a descriptive data analysis will be performed with a cleaning of the database, an analysis of the categorical variables using frequencies and percentages (%) for each category.

#### Analysis of preliminary effectiveness

The preliminary clinical effectiveness of the interventions will be conducted on an intention-to-treat (ITT) approach with the ADHD-RS-IV total score as a primary outcome (McCoy, 2017). Linear mixed-effects regressions with restricted maximum likelihood (REML) will be employed. REML accounts for the correlation between repeated measures for each individual and provides less biased estimates of variance parameters, which is particularly useful when dealing with small sample sizes or unbalanced data (Egbewale et al., 2014). No imputation of missing data will be performed since it has been reported that multiple imputation is unnecessary for certain types of missing data (missing completely at random, missing at random, and missing not at random) when conducting longitudinal mixed model analysis (Twisk et al., 2013). Unstandardized regression coefficients (B) and 95% confidence intervals (95% CIs) will be computed for the ‘group x time’ interaction between groups at post-treatment and 5-month follow-up assessments. Cohen’s d will be calculated for each pairwise comparison, using the pooled baseline standard deviation to weigh the differences in the pre-post mean values and adjust for population estimates (Morris, 2008). Cohen’s d effect size interpretation will use the classical cutoffs of 0.20 = small, 0.50 = medium, and 0.80 = large. The same statistical procedure will be applied to analyze all secondary clinical endpoints. To control for false rate discovery, the Benjamini–Hochberg correction for multiple comparisons will be utilized (Glickman et al., 2014).

The analyses will be replicated using a “completers” approach, including all participants who completed the study, and a per-protocol approach, considering only those patients who attended at least 75% of the sessions (6 out of 12).

Additionally, to assess the clinical significance of improvement in the primary outcome (ADHD-RS-IV), the participants will be allocated into the categories of “responders” and “non-responders” according to whether they obtains a ≥40% reduction in ADHD-RS-IV total score (Weiss et al., 2019). This classification will be used to calculate the number-needed-to-treat (NNT) for each treatment compared to the others. The NNT estimates the number of patients needing treatment with a new proposed treatment (instead of the control comparison treatment) for one additional patient to benefit. A 95% CI for each NNT will be computed, making the findings more clinically meaningful to practitioners.

Furthermore, potential predictors of treatment response in each intervention arm will be explored. T-tests and χ2-tests will be performed to assess any baseline differences in sociodemographic and/or clinical variables between responders and non-responders within the M4H, CT and MCT arms at the end of the intervention and at 5-month follow-up.

Finally, baseline differences between participants who completed all study assessments and those who did not will be evaluated (using t-tests and χ2-tests) to detect any potential attrition bias.

#### Feasibility analyses

The refusal rate will be assessed using the percentage of individuals who decline to participate in the study when approached or invited to do so. Retention will be evaluated through evaluation of the number of group sessions attended (in M4H and MCT arms), number of online sessions attended (in CT and MCT arms) and level of adherence to home practice (completion percentage of proposed homework). The proportion of participants who experienced positive outcomes (changing at least one level of severity in the ADHD-RS scale) vs. those who did not will also be evaluated (failure/success rate). The dropout rate will also be imputed by considering the number of participants who discontinue the study before its conclusion. The percentage of participants’ perceived adverse effects (e.g., anxiety, headaches, dizziness) related to the interventions will also be reported. Level of satisfaction with the received intervention will be evaluated at the end of treatment through MCT, M4H and CT parents and children satisfaction questionnaires, and through CEQ. Differences between active arms (M4H, CT and MCT) regarding all aforementioned feasibility measures, will be assessed using ANOVA and *post hoc* tests.

#### Mediation analyses

We will calculate pre-post change scores for all process measures in the study and pre-follow-up change scores for primary and secondary outcomes. Bivariate Pearson correlations will be computed between the pre-post change scores for the process variables and the pre-follow-up change scores for the clinical endpoints to identify statistically significant relationships. Path analyses will be used to examine the direct and indirect associations between the treatment conditions (i.e., M4H, CT, MCT, and TAU as independent variables), process variables (mediators), and primary and secondary outcomes (dependent variables). This statistical approach considers temporality, which enhances the ability to establish causal conclusions. Regression coefficients (B) of bias-corrected bootstrapped indirect effects will be calculated, along with their standard errors (SEs) and 95% confidence intervals (CIs; Lockhart et al., 2011). Indirect effects will be considered statistically significant when the 95% CI does not include 0. Participants with missing data will be excluded from this analysis.

## Discussion

The NeuroMind study will evaluate the preliminary effectiveness and feasibility of adding the M4H program, a Computerized CT program and, particularly, a combination of both approaches (i.e., MCT) to TAU in children with ADHD. In order to determine the mechanisms of action of these interventions, we will also evaluate potential process measures of these interventions (i.e., mindfulness and emotional regulation) with evidence of alteration in ADHD and that are potentially modifiable by the proposed interventions. Furthermore, potential predictors of treatment response in each intervention arm will be explored to identify preliminarily potential subsets of patients prone to respond to each treatment arm.

One previous study of the group evidenced the short-term efficacy (i.e., post-intervention) compared to TAU of the M4H (Huguet, 2021) and there is mounting evidence that CT interventions could be used as effective ways for treating ADHD (Cortese et al., 2015; Caye et al., 2019; Fernández-Daza, 2019; Drechsler et al., 2020; Veloso et al., 2020). Particularly, Bursky et al. (2022) and Sanchez-Lara et al. (2023) suggest the potential benefits of combining CT and MBIs in the treatment of depression and in the improvement of cognition, mood and quality of life of older adults, respectively. However, as far as we know, no study assessed the benefits of combining both approaches in children with ADHD.

By addressing at the same time the cognitive (mainly through CT but also using M4H) and emotional aspects (particularly through M4H) of ADHD, the combined program MCT theoretically may offer a comprehensive and more effective approach to improving ADHD symptoms and associated difficulties. This study will be the first to assess the potential utility and feasibility of such a combination, along with evaluating if the clinical benefits of the three proposed approaches can be maintained at a 5-month follow-up.

Clinically relevant effects are expected to be observed in all three treatment arms compared to TAU, both at post-intervention and at 5-month follow-up. One of the challenges of this project may be the high likelihood of dropouts and non-compliance, especially in the MCT program group which combines two different interventions. To reduce the impact of these factors on our findings, we will perform different sensitivity analyses (ITT, completers, per protocol analysis). Another issue is the absence of blinding for participants and therapists, which is a common source of bias in any RCT involving non-pharmacological interventions. Despite having a weekly assessment to monitor the adherence to mindfulness homeworks, some participants/parents may exaggerate children’s practice. Moreover, the small sample size of this pilot study will hinder the probability of detecting statistically significant differences between active arms on primary and secondary outcomes. However, it is hypothesized that the preliminary effectiveness in the short-and medium-term of the three programs will be demonstrated, with larger effect sizes for M4H and MCT programs in the primary outcome of ADHD symptomatology. According to this hypothesis, the NeuroMind study could be a preliminary basis for conducting an RCT with a larger sample to validate these interventions to be included in clinical practice. A third limitation of the NeuroMind study could be that all three interventions are not totally comparable in therapeutic dose, because the M4H and MCT programs involve more therapist time than the CT program, which is mostly self-administered. The M4H and MCT programs require more involvement from therapists than the CT program, which leans more toward self-administration. Specifically, the M4H and MCT programs involve a weekly in-person session for children lasting 1.25 h, six weekly at-home children’s sessions lasting around 10 min each, and three 1.25-h sessions involving both parents and children throughout the intervention period. On the other hand, the CT program consists of only six at-home weekly sessions for children lasting approximately 10 min each, along with three 1.25-h sessions that include both parents and children throughout the intervention. One possible outcome is that the MCT intervention is more effective than the other two compared to TAU. However, this intervention might also be more costly and difficult to implement, as it would need more resources. Further, well-powered studies should assess the differential cost-effectiveness of the interventions under the evaluated scenarios. In this regard, if the MCT intervention were more costly but saved resources (and was more cost-effective), it would be a good justification for its implementation in our healthcare system.

A potential drawback of implementing the cognitive training intervention at home is that participants and their parents need to consistently remember to carry out the sessions daily. We will provide weekly phone call supervision for this group to address this challenge. Additionally, we will conduct in-person psychoeducational sessions during weeks 1, 4, and 8. These sessions are designed to reinforce compliance with the treatment plan to enhance treatment adherence.

Regarding the strengths of the NeuroMind study, first of all, the feasibility and the preliminary short-and medium-term effects of the evaluated treatments will be assessed, including a wide range of measures to assess ADHD symptoms and also EF. The fact that the three interventions are in online format (CT, MCT) and/or in group format (CT, M4H, MCT) could also be considered a strength. On the one hand, these formats can make them more cost-effective than interventions in only on-site and/or individual formats. On the other hand, the online format of the CT program has more flexibility and scalability than on-site interventions and other online interventions since its online sessions can be done at any time of the day.

Finally, especially the combination of both approaches, adding to some of their ADHD Treatment-As-Usual (non-treatment, psychopharmacology, psychiatric monitoring and/or psychological monitoring) could be a strength because it can be a more effective treatment than CT only program or M4H only program, and also all three interventions could be more effective to children with ADHD that do not respond or show adverse effects to ADHD Treatment-As-Usual.

If treatments evaluated in the NeuroMind study are shown to be effective (compared to TAU) and feasible, a full RCT will be conducted in the future to assess effectiveness in a larger sample of participants. If the M4H, CT and MCT programs are definitively validated, then they should be incorporated into clinical practice, increasing our non-pharmacological treatment options for approaching ADHD.

### Ethics issues and dissemination

Prior to the start of the study, the study protocol was presented to the Sant Joan de Déu Foundation Ethics Committee and, following the protocol of good research practice, the study protocol has been registered at ClinicalTrials.gov (Trial registration number: NCT05937347).

Participants who meet the inclusion/exclusion criteria and whose parents or guardians will sign informed consent to participate in the research will be included. Verbal assent from the child will also be sought. The research team will ensure that data will be collected anonymously. Patients will be identified with a numerical code that will be known only to the principal investigator concerning the patient’s identity. Personal data will be replaced by codes and data that must necessarily be collected by clinicians (e.g., age, sex, medical history) and will be stored separately from other data and will only be available to the persons responsible for the development of the project, always protecting the right to privacy of the participants at all times. The data will always be treated confidentially and used only for this research by the Principal Investigator (AH-M) and the research team, according to the regulation (EU) 2016/679 of the European Parliament and the Council of 27 April 2016 on Data Protection (GDPR) and to the national implementing regulations. The controller of the database will be the SJDTLL Center. The database will be located in a folder on the SJDTLL secure and restricted access network, only the principal investigator and collaborating researchers of the center will be able to access it. The Organic Law 3/2018, of 5 December, on the Protection of Personal Data and Guarantee of Digital Rights will be followed.

Concerning the NeuronUP digital platform, patients will be identified with their relevant study-specific numerical code and no personally identifiable data will be shared with the NeuronUP® platform. NeuronUP S. L. will collect the platform usage data and will form part of an anonymised database that will be subject to analysis by the company to improve the platform itself, as indicated in the informed consent.

### Blinding

Randomization and group allocation will be completely masked for the study assessors. Study participants will be asked not to communicate with the assessor about the treatment received. The study participants will be provided with a summary of evidence of the treatments in the NeuroMind study. As is usual in non-pharmacological trials, neither participants nor the therapists can be blinded to treatment allocation.

### Forecast execution dates

Initial recruitment of patients: September 2023.

Finalization of patient recruitment: March 2024.

Finalization of patient monitoring period: September 2024.

Publication of results: June 2025.

## Ethics statement

The studies involving humans were approved by Sant Joan de Déu Foundation Ethics Committee. The studies were conducted in accordance with the local legislation and institutional requirements. Written informed consent for participation in this study was provided by the participants’ legal guardians/next of kin.

## Author contributions

TB-A: Writing – original draft, Writing – review & editing, Conceptualization, Visualization. ER-C: Visualization, Writing – review & editing. VP-G: Visualization, Writing – review & editing. PV-B: Visualization, Writing – review & editing. AC: Visualization, Writing – review & editing. JL: Methodology, Visualization, Writing – review & editing. JS: Visualization, Writing – review & editing. AF-S: Conceptualization, Funding acquisition, Methodology, Project administration, Supervision, Visualization, Writing – original draft, Writing – review & editing. AH: Conceptualization, Funding acquisition, Methodology, Project administration, Resources, Supervision, Visualization, Writing – original draft, Writing – review & editing.

## Footnotes

^1^ www.sealedenvelope.com
